# GSB: GNGS and SAG-BiGRU network for malware dynamic detection

**DOI:** 10.1371/journal.pone.0298809

**Published:** 2024-04-18

**Authors:** Zhanhui Hu, Guangzhong Liu, Xinyu Xiang, Yanping Li, Siqing Zhuang

**Affiliations:** 1 College of Information Engineering, Shanghai Maritime University, Shanghai, China; 2 College of Artificial Intelligence, Jiangxi University of Technology, Jiangxi, China; 3 College of Merchant Marine, Shanghai Maritime University, Shanghai, China; University of the West of Scotland, UNITED KINGDOM

## Abstract

With the rapid development of the Internet, the continuous increase of malware and its variants have brought greatly challenges for cyber security. Due to the imbalance of the data distribution, the research on malware detection focuses on the accuracy of the whole data sample, while ignoring the detection rate of the minority categories’ malware. In the dataset sample, the normal data samples account for the majority, while the attacks’ malware accounts for the minority. However, the minority categories’ attacks will bring great losses to countries, enterprises, or individuals. For solving the problem, this study proposed the GNGS algorithm to construct a new balance dataset for the model algorithm to pay more attention to the feature learning of the minority attacks’ malware to improve the detection rate of attacks’ malware. The traditional malware detection method is highly dependent on professional knowledge and static analysis, so we used the Self-Attention with Gate mechanism (SAG) based on the Transformer to carry out feature extraction between the local and global features and filter irrelevant noise information, then extracted the long-distance dependency temporal sequence features by the BiGRU network, and obtained the classification results through the SoftMax classifier. In the study, we used the Alibaba Cloud dataset for malware multi-classification. Compared the GSB deep learning network model with other current studies, the experimental results showed that the Gaussian noise generation strategy (GNGS) could solve the unbalanced distribution of minority categories’ malware and the SAG-BiGRU algorithm obtained the accuracy rate of 88.7% on the eight-classification, which has better performance than other existing algorithms, and the GSB model also has a good effect on the NSL-KDD dataset, which showed the GSB model is effective for other network intrusion detection.

## 1. Introduction

Cyber security risks brought by malware threats everywhere range from personal information disclosure to corporate data theft to even vital national secrets. We classify the malware into viruses, worms, trojans, ransomware, backdoors, etc., according to their different functions. The malware analysis techniques include static analysis, dynamic behavior analysis, and hybrid analysis [1, 2]. Static analysis extracts valuable information from binaries without executing the software to classify [3, 4]. Dynamic analysis is executing the malware for classification in an isolated environment by monitoring its behavior, interaction with the system, and impact on the system [5, 6]. Both static analysis and dynamic analysis of malware have their advantages and disadvantages. Static analysis may be fooled by software encryption and obturation technology, leading to detection failure [7]. Dynamic analysis requires more computer resources and provides the target system environment of malware to trigger malicious behaviors of malware. The conventional antivirus engine detects malware based on the signature, runtime behavior, and heuristics.

With the development of artificial intelligence technology, machine learning and deep learning have made many remarkable research achievements in computer vision, natural language processing, and other fields [8, 9]. The scholars continue to explore their applications and breakthroughs in malware detection. However, there are some problems with current malware detection technology [10–12].

1. Traditional malware detection methods rely on professional knowledge to extract features and lack good algorithms for training model to extract features.
2. Some machine learning and deep learning, such as CNN and RNN, made some achievements, but for some large datasets, the generalization ability is poor, and can’t be calculated in parallel.
3. Ma et al. [13] only detected the malware, and didn’t classify the malware family, and ignored the detection rate of the minority categories’ malware.
4. The category distribution of the dataset is unbalanced, with the edge distribution of minority categories. Machine learning isn’t enough to learn the features of minority categories, and the detection rate is mediocre.

### 1.1 Innovations and contributions

Due to the imbalance of malware dataset, the normal data samples account for much more compared to the minority categories, and the model focuses on the feature learning of the whole data sample, while ignoring the feature learning of the minority categories’ malware. The SMOTE algorithm is the most common sampling algorithm, but the SMOTE algorithm also has some shortcomings that can’t produce the new data sampling. The new synthetic samples tend to the majority categories sample area, being noise samples, and the new samples are easy to cause the overfitting of the model. The classification performance for the traditional machine learning algorithms on unbalanced datasets is poor and can’t identify the minority attack samples well, which usually cause big loss for the users. To solve the problems, we proposed the Gaussian Noise Generation Strategy (GNGS) algorithm and SAG-BiGRU network model for the malware detection. The Innovations and contributions of the paper is shown in Table 1.

**Table 1:** Innovations and contributions of the paper.

a) Most researchers can’t detect the minority categories better. In this paper, we proposed the Gaussian Noise Generation Strategy (GNGS) algorithm to construct a new balance data set for the first time in the field of cyber security and could perform multiple classifications of data sets. The precision of minority categories is much higher than other sampling methods, such as random sampling and Smote sampling which generated noise and caused the overfitting of the model to reduce the precision of malware detection. However, the GNGS algorithm could generate new sample points and increase the robustness and generalization ability of the model.
b) Most model algorithms can’t learn the temporal sequence and can’t compute in parallel, such as RF, RNN, and CNN, whose generalization ability is poor for some large datasets. This paper proposed the SAG deep learning model to learn the temporal sequence features, which is first applied to the Alibaba cloud dataset.
c) We proposed the Self-Attention with Gate mechanism (SAG), which could effectively filter irrelevant noise information, extract the key features, and extract the long-distance dependency temporal sequence features of data by the BiGRU network, which is better than other studies that have been done.
d) The SAG-BiGRU deep learning models have a high convergence efficiency and short time consumption compared with other algorithm models such as CNN, Random Forest, and the like. The GSB model is also better than previous studies on the NSL-KDD dataset.

## 2. Literature related work

We divide the malware detection methods into traditional methods and machine learning methods. Traditional methods strongly rely on professional knowledge to extract effective features but need huge human cost and time input, especially when the amount of malware detected is large. Although the traditional manual analysis method has high accuracy, it is extremely inefficient and costly. We divide the machine learning models into traditional machine learning models and deep learning models.

Literature [14, 15] proposed a malware detection method that combined malware visualization technology with the CNN model. Ding et al. [16] presented a static detection method, which directly extracted a bytecode file from an Android APK file, converted the bytecode file into a two-dimensional matrix, and then used the CNN algorithm to train the detection model. Gupta and Rani [17] have designed two methods of the weighted voting strategy based on ensemble learning to improve the performance of malware detection. Liu et al. [18] proposed the data visualization and adversarial training to detect the malware and its variants. Zhong and Gu [19] proposed a Multi-Level Deep Learning System that used the tree structure model to focus on learning a specific data distribution of a particular group.

Venkatraman et al. [20] proposed a unified hybrid deep learning and visualization approach for malware detection, which aimed to use the image techniques for detecting suspicious behavior and investigate the application of a hybrid image-based approach. Surendran et al. [21] proposed the Tree Augmented Naive Bayes-based hybrid malware detection mechanism for identifying whether the application is malicious. Jeon et al. [22] used the dynamic analysis for IoT malware detection, which used the CNN model and analyzed IoT malware dynamically in the nested cloud environment.

Lu et al. [23] proposed a hybrid deep learning model for malware detection, which combined a Deep Belief Network and Gate Recurrent Unit. Alzaylaee et al. [24] proposed the malicious detection of Android applications based on the deep learning system through dynamic analysis. Amer et al. [25] introduced the word embedding to understand the contextual relationship between API functions and call sequences. Arora et al. [26] proposed the PermPair detection model, which compared the graphs for malware and normal samples through extracting the permission pairs from the manifest file of an application. Khan et al. [27] utilized GoogleNet and ResNet models to identify the malware and obtained a testing accuracy of 74.5% on GoogleNet.

In the static detection of malware, the deep learning method represented by CNN and RNN can achieve a better software classification effect with the help of static feature engineering. However, due to the interference of software encapsulation, obfuscation, and other technologies, it is tough to extract static features to catch up with the evolution of malware. The dynamic detection of malware extracts the features generated in the running process of software, and uses the sandbox technology to capture the behavior information of malware during running, such as creating files, deleting files, encrypting information, creating threads, interacting with the registry, obtaining virtual address space, and so on. The API call sequence is usually the most crucial dynamic feature in malware detection and classification.

In recent years, it has proposed the concept of attention mechanism that has been integrated into deep learning models. Jindal et al. [28] got the design inspiration from file classification, combined CNN, and BiLSTM network, and proposed a neural network model Neurlex for dynamic malware detection without feature engineering, whose limitation is that it requires the execution of the code to determine whether the code is malicious or not. Yoo et al. [29] proposed a machine learning-based hybrid decision model, which combined a random forest and a deep learning model to determine malware and benign files, whose experimental result achieved an 85.1% detection rate. Literature [30] proposed the Adaptive Malware Analysis Dynamic Machine Learning (AMDML) algorithm based on a rule called federated learning, which obtained better accuracy compared to the machine learning, but the accuracy is still not high and didn’t classify the malware, and the study will design a flexible environment for applications. Mazhar et al. [31] proposed image-based malware classification using the VGG19 network and spatial convolutional attention, but didn’t deal with the imbalance of the data categories and lacked the exploration of the scale data, the feature engineering, and implement algorithm parallelization calculation.

Xu et al. [32] used a One-hot coding method to classify malicious codes according to API sequences and other features, and the accuracy of their CNN and LSTM hybrid models. However, this method didn’t consider the robustness of the model, that is, in the face of the obfuscation, shell, and other counter-detection techniques commonly used by malware developers. Literature [33] used a novel malware classification method that captures suspected operations in a variety of discrete size image features based on CNN to identify such IoT device malware families. Hamad Naeem et al. [34] proposed a platform-independent malware detection and classification scheme with process-based volatile memory forensics and a deep stacked ensemble based on the structural and statistical image textural analysis by the CNN, which achieved a good classification effect but a large number of features caused the long training time.

For the above literature, they only considered detecting whether the software was malicious or not. Many studies didn’t consider the imbalance of training datasets and the edge distribution of minority categories will cause the model algorithm to disregard the characteristics of the minority data. Many studies lacked the exploration of the scale data, and algorithm model didn’t perform the parallelization calculation which caused the long training time.

## 3. Model design

**Fig 1:**
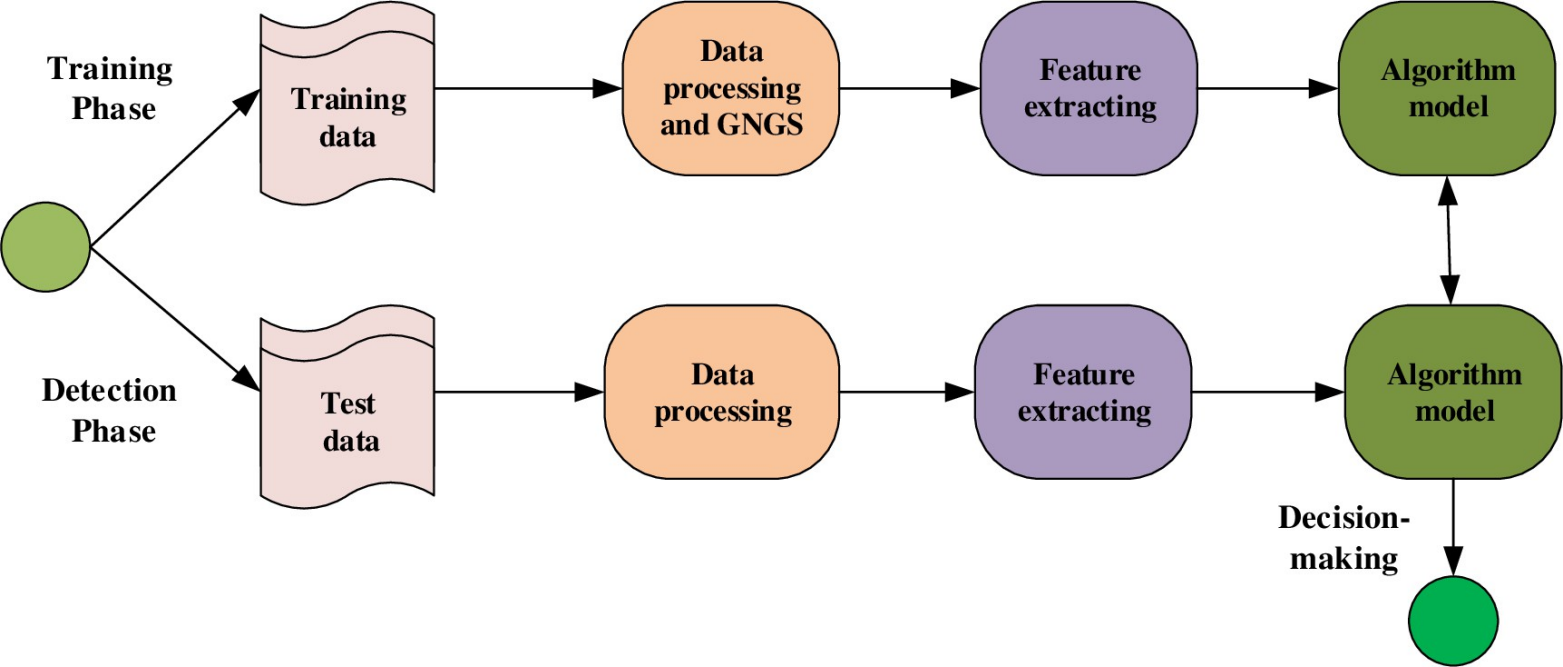
Framework of the method.

This part will introduce the framework of the method proposed in this study, as shown in Fig 1, including data preprocessing and GNGS for constructing new data, feature extraction, and algorithm model construction. Since API calls are temporal sequences, we can handle the malware classification in BiGRU processing the temporal sequence. Malicious snippets of code in an API sequence are not necessarily contiguous, so added the Transformer to the train model for learning malicious API sequences.

After handling the data by de-duplicating, concatenating into a long string according to the thread number for data processing of every file, we use the Random Gaussian algorithm to learn the distribution of minority categories samples data through the generator to build a balanced data set.

**Fig 2:**
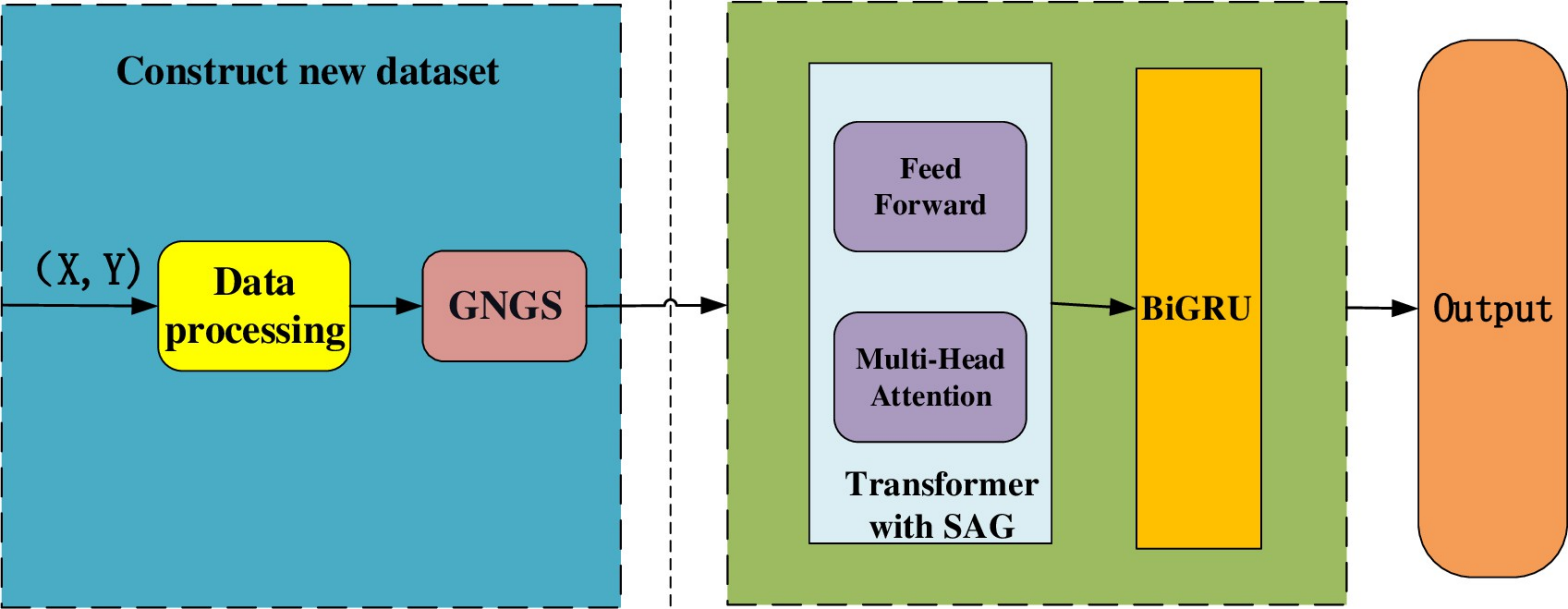
The framework of the model.

Input the new dataset into the Transformer with SAG module to establish connections between different features, extract richer feature information and filter the irrelevant noise information. After that, the data is input into the BiGRU neural network to obtain the relationship between the front and back features to retain their temporal sequence information. Finally, classify the output by the Softmax classifier. This model draws on the advantages of various models and can simultaneously consider the connection between different features and the temporal sequence information of features. The framework of this model is shown in Fig 2.

### 3.1 Data set

The experimental dataset in this study is provided by Alibaba Cloud security malicious program detection [35], which contains the latest attack types, and the data error rate is close to zero. The dataset is suitable for malware detection research. The data comes from the API instruction sequence recorded by the desensitized Windows binary executable program (PE file) after simulation running in the sandbox program. There are seven types of malware: ransomware, mining program, DDoS Trojan, worm virus, infection virus, backdoor program, and Trojan program.

In this study, the data records more than 89 million API calls in 13,877 binaries with a data file size of 2.7GiB. The average number of API calls per file is 6466, with the highest number of calls being 888,204. The data set consists of file number file_id, file label, API name, thread number, and sequence number of API calling in the thread index. Taking file_id 2 as an example, the data sample is shown in Table 2, in which each file has multiple API calls, and there may be some sequence relationship between APIs, but there is no sequence relationship between different threads. The sequence number Index in Tid of the same thread number from small to large represents the sequence relationship of calls. The Index is the global order of execution of a single file in the sandbox. The same thread or different threads may execute API many times on an Index, but the Index inside Tid with the same thread number is in a certain order.

**Table 2:** Dataset sample.

file_id	label	api	Tid	Index
2	2	GetSystemTimeAsFileTime	2320	0
2	2	SetUnhandledExceptionFilter	2320	1
2	2	NtAllocateVirtualMemory	2320	2
2	2	WSAStartup	2320	3
2	2	GetSystemTimeAsFileTime	2320	4
…	…	…	…	…
2	2	NtClose	2604	200
2	2	NtClose	2604	201
2	2	RtlRemoveVectored ExceptionHandler	2604	202
2	2	NtTerminateProcess	2604	203

### 3.2 Data preprocessing

Each file in the original data calls multiple APIs in the process of dynamic execution and may be called by different threads. In this paper, we handle the data by concatenating. That is, the API called by the same file is concatenated into a long string according to the thread number and the sequence of intra-thread calls. However, the provider of the dataset states that there is no sequential relationship between the different threads. We constructed an additional feature to represent the thread number to reflect the relationship between the input data.

Since there are a large number of adjacent repeating APIs in the API sequence, in the experiment, we de-duplicated the API sequence and converted the API sequence into the sequence of numbers, as shown in Table 3, by using different numbers to represent the API and the relevant data preprocessing is shown in Algorithm 1. Since the API sequence length corresponding to each file is different, according to the data format requirements of the model input, we need to select a specified sequence length and truncate for the long API sequence length exceeding.

**Table 3:** Sequence converted from API to digital number.

API sequence	Digital number
GetSystemTimeAsFileTime	18
SetUnhandledExceptionFilter	91
NtAllocateVirtualMemory	11
WSAStartup	97
GetSystemTimeAsFileTime	18
GetSystemInfo	56
SetErrorMode	37
LdrGetDllHandle	25
LdrGetProcedureAddress	1
LdrGetProcedureAddress	1
LdrGetProcedureAddres	1

**Algorithm 1:** De-duplicate and sorte api and build mappings between apis and numbers .

1:df = pd.read_csv(’security_train.csv’,low_memory=False)

2: grouped = df.sort_values([’file_id’, ’tid’, ’index’]).groupby(’file_id’) #Sort and group

#api columns are converted to numbers

3: def api_to_num(api_seq):

4:  api_set = sorted(set(api_seq)) #api columns are de-duplicated and sorted

5:  api_num_seq = [api_to_num_dict[api] for api in api_seq] # Build mappings

6:   return api_num_seq

#Each group and the results are stored in a new column

7:  df_agg = grouped.agg({’api’: ’ ’.join, ’label’: list}).reset_index()

8:  df_agg[’api_num’] = df_agg[’api’].apply(lambda x: api_to_num(x))

9:  api_num_list = df_agg[’api_num’].tolist()

10: label_list = df_agg[’label’].tolist()

11: label_list = [l[0] for l in label_list]

#### 3.2.1 Data processing and GNGS

After converting the API call sequence into a number sequence and word vector, input the model for training. Take a file as an example to show the API called by the infectious virus with file_id 1 and label 5 during dynamic execution in the sandbox, and the call sequence of the first 20 APIs is splicing according to thread number and API call order within the thread: “LdrLoadDll LdrGetProcedureAddress LdrGetProcedureAddress LdrGetProcedureAddress LdrGetProcedureAddress LdrGetProcedureAddress LdrGetProcedureAddress LdrGetProcedureAddress LdrGetProcedureAddress LdrGetProcedureAddress NtCreateMutant NtClose NtCreateFile NtWriteFile NtClose CreateProcessInternal NtClose NtClose LdrUnloadDll NtAllocateVirtualMemory…”. For example, the call of LdrLoadDLL makes the function of the infectious virus difficult to understand by reverse analysis of engineers because LdrLoadDLL is an undisclosed API in the Windows ntdll.dll code base. Windows system provides Windows developers with many code libraries to access its functions, such as kernel32.dll and ntdll.dll, kernel32.dll code library provides all the basic core functions of the program, including reading files and writing files, etc. ntdll.dll is the back-end code base of kernel32.dll to support the basic functions of kernel32.dll. Due to the different functions and purposes of each software, the behavior of the dynamic execution process is diverse, and the length of the API call sequence is also distinct. Even more, some malware is created by benign software inserting malicious code snippets, and some malware uses obfuscation techniques to repeatedly call a large number of useless APIs. In the data set, the average number of API calls per file is 6466, the maximum number of API calls is 888204, and the average length of API calls is much longer than the sequence length for the model text processing. To make full use of the information in the API call sequence, and take into account the computational power, we need to truncate or fill sequences of different lengths for training and testing. We will set the maximum length to 780, truncate the sequences that exceed the maximum length, and fill the sequences that are less than the maximum length with -1, as shown in Algorithm 2. To convert a string API call sequence into a numeric vector, we first need to tokenize the above text. Each file corresponds to a sequence of strings representing the API to call.

**Algorithm 2:** Trimmed and padded for api.

1: def trim_and_pad(api_seq, seq_len=780):

2:    if len(api_seq) > seq_len:

3:       return api_seq[:seq_len]

4: else:

5:       return api_seq + [–1] * (seq_len - len(api_seq))

6: api_num_trimmed_list = [trim_and_pad(seq) for seq in api_num_list]

7: api_num_tensor_list = [torch.tensor(seq, dtype=torch.long) for seq in api_num_trimmed_list]

8:  padded_api_num_tensor = torch.nn.utils.rnn.pad_sequence(api_num_tensor_list, batch_first=True, padding_value=-1)

9:  input_ids = padded_api_num_tensor

10: attention_mask = input_ids != -1

11: labels = torch.tensor(label_list)

12: print(input_ids.shape)

13: print(labels.shape)

Most existing studies use the Smote algorithm to deal with the imbalance of the dataset. The Smote algorithm adopts an interpolation method when synthesizing new samples. For minority category sample a, the algorithm randomly selects sample b from its nearest neighbor, and then randomly selects a point on the line between a and b as the newly synthesized minority category sample.

#### 3.2.2 GNGS

However, the Smote algorithm also has some shortcomings which are sensitive to noise and don’t have a good effect on the large-scale samples, and the synthetic sample is easy to be in the majority of class sample areas, forming noise samples. Finally, it causes the overfitting of the model to reduce the precision of malware detection. In order to compensate for the shortcomings of Smote, we proposed the GNGS algorithm to deal with unbalanced minority attack data categories. The GNGS algorithm is not only used for clustering but also for estimating probability density. More importantly, the GNGS algorithm can generate new sample points and increase the robustness and generalization ability of the model.

After the data processing, the data samples are perturbed by the Gaussian Noise Generation Strategy (GNGS). In this process, we add the Random Gaussian noise to each continuous feature, with its standard deviation proportional to the original feature value, as shown in Algorithm 3. The GNGS expanded and homogenized the data set, realizing the processing of the unbalanced dataset, which generated a new dataset with a more balanced distribution of categories. Random.gauss (mu,sigma) is a Gaussian random number generator, Where mu is the mean value and sigma is the variance. In the experiment, the mean value is 0, and the variance is 0.01. In this method, the generated new sample features will be highly homogenous, and the training model will pay more attention to the useful minority category features.

**Algorithm 3:** Gaussian Noise Generation Strategy.

1: def generate_new_sample(row, std_dev=0.01):

2: new_row = row.copy()

3: for feature in new_row.index:

4:   if isinstance(new_row[feature], (int, float)):

5:     new_row[feature] += random.gauss(0, std_dev * new_row[feature])

6:  return new_row

7: DDoS_indices = label_data[label_data["label"] == "DDoS"].index

8: new_samples_count = 2900 # Generate the number of new samples generate

9: new_DDoS_samples = []

10: new_DDoS_labels = [] # Generate a new DDoS sample

11: for _ in range(new_samples_count):

12:   random_row = DDoS_data.sample()

13:   index = random_row.index[0]

14:   new_sample = generate_new_sample(random_row.iloc[0])

15:   new_DDoS_samples.append(new_sample)

16:   new_DDoS_labels.append("DDoS")

### 3.3 SAG unit based on transformer

Transformer is a deep learning model launched in 2017, mainly used in the natural language processing (NLP) field [36, 37]. Transformer is designed to process sequential data such as translation and text classification. Compared with RNN, the Transformer can support parallelization, thus greatly reducing the training time [38, 39].

**Fig 3:**
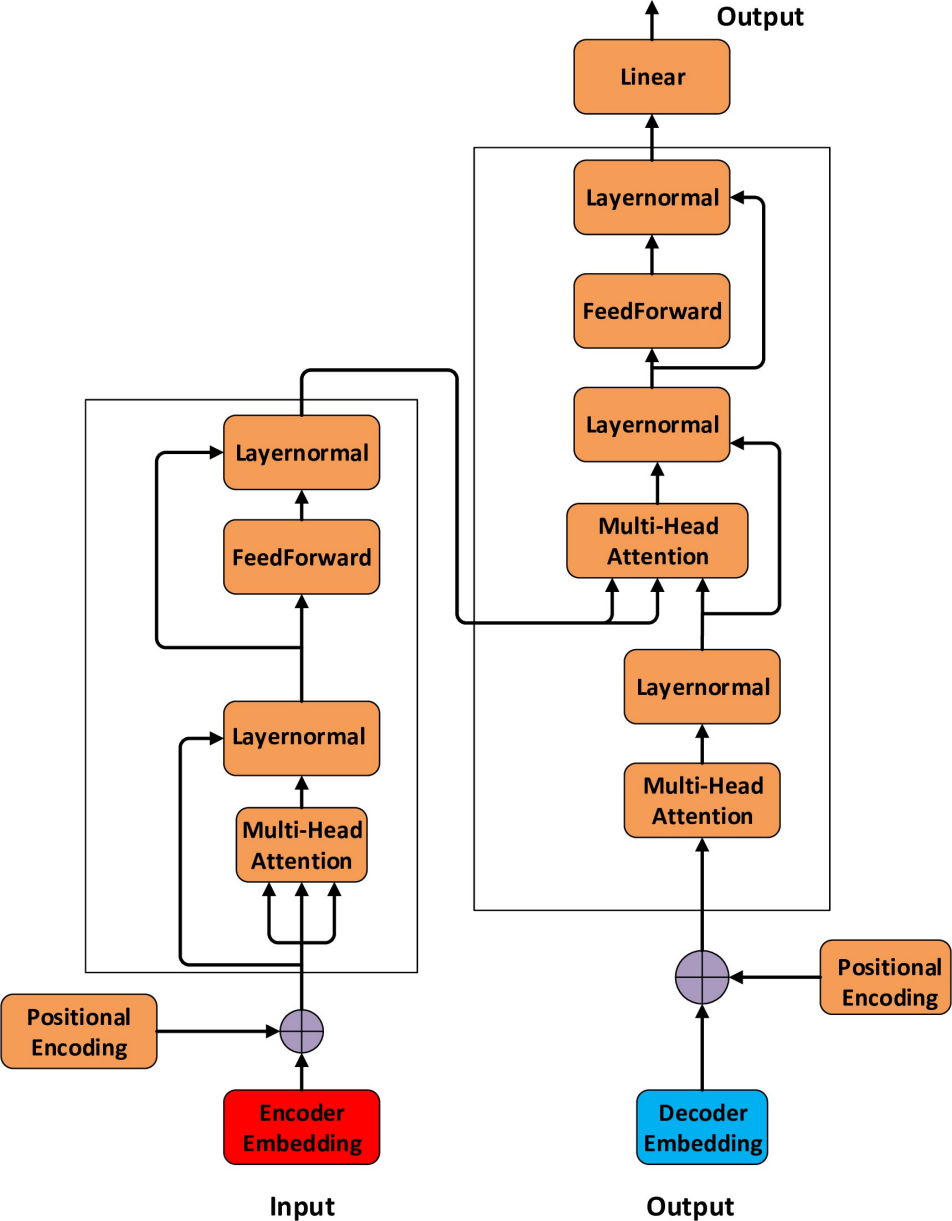
The framework of the transformer.

The transformer includes the encoder-decoder architecture, as shown in Fig 3. An encoder consists of a set of coding layers that iterate through the input layer after layer, and a decoder consists of decoding layers that perform the same action on the output. Each encoder consists of a Self-Attention layer and a Feed Forward Network. Word vectors flow through the two sublayers of the encoder.

#### 3.3.1 Attention mechanism

In all the APIs invoked, it is not hard to see that each has the same impact on the dynamic behavior of the software for each category. However, Attention mechanisms can extract malicious features that are crucial to behavior classification at a single API level. Taking the sequence "NtCreateFile NtWriteFile NtClose" as an example, calculate the attention score for the first word NtCreateFile. We need to calculate the correlation score of each of the other words to NtCreateFile, which determines how much attention should be given to the other words when encoding the current word.

The attention mechanism uses dot product attention, which has three inputs: Query, Key, and Value. Use Query and Key to calculate the weight score assigned to each value, and then the weight and value are weighted to obtain the output. Using dot product attention can perform parallel operations, reducing training time. Its calculation Formula (1) is as follows.


$$Attention\;(Q,K,V)=soft\;\max\;(\frac{QK^{T}}{\sqrt{d_{k}}})$$ (1)

In the Formula (1), Q, K, and V respectively represent Query, Key, and Value, and d_k_ is the dimension of Key. To enrich the extracted features, this study uses the structure of multi-head attention, whose Formulas are as follows (2–4).


$$Q_{i}=QW_{i}^{Q},K_{i}=KW_{i}^{K},V_{i}=VW_{i}^{V}\;i=1,\ldots ,n$$ (2)

$$head_{i}=Attention\;(Q_{i},K_{i},V_{i})\;i=1,\ldots ,n$$ (3)

$$MultiHead\;(Q,K,V)=concat\;(head_{1}\ldots head_{n})W^{o}$$ (4)

#### 3.3.2 SAG unit

**Fig 4:**
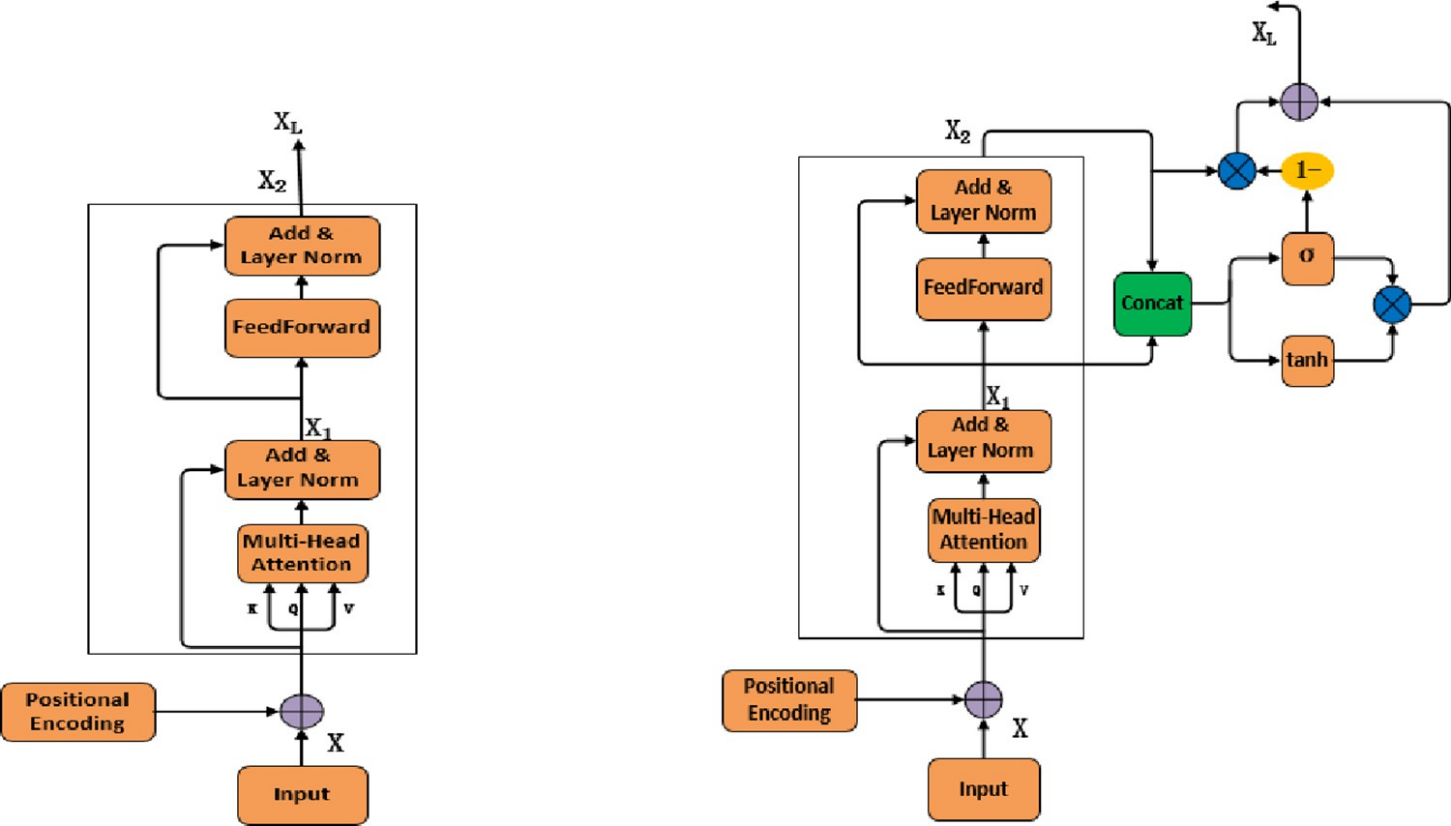
Self-Attention with Gate mechanism (SAG) model for encoder. (a) Self-Attention in Transformer and (b) Self-Attention with Gate mechanism.

Transformer uses a self-attention layer to encode understanding of related words into the current word. The Multi-head attention mechanism extends the model’s ability to focus on different locations. We use an additional position encoder to reflect the position between API sequences, generate the position vector, and add the position vector to the word vector, which can better represent the distance relationship between words. We proposed the Self-Attention with Gate mechanism (SAG) to extract the key feature information and effectively filter irrelevant noise information, as shown in the Fig 4.

Input X into the SAG unit to learn the more important and rich features information and filter the invalid information by the Gate mechanism. The feature matrix formula are shown in the Eq (5–10).


$$X_{1}=MHA(Q_{X},K_{X},V_{X})=concat(head_{1}\ldots head_{n})W^{o}$$ (5)

$$X_{2}=FFN(X_{1})=\max(0,X_{1}W_{1}+b_{1})W_{2}+b_{2}$$ (6)

$$X_{3}=concat(X_{2},X_{1})$$ (7)

$$X_{t}=\tanh(X_{3})$$ (8)

$$X_{s}=sigmoid(X_{3})$$ (9)

$$X^{\left(L\right)}=X_{t}*X_{s}+(1-X_{s})*X_{2}$$ (10)

Where W_1_, W_2_ represent the weight and b_1_, b_2_ represent bias variable. We obtained the feature matrix *X*_*L*_ by the gate mechanism layer. Where *X*_*L*_ is the final output feature matrix through the SAG unit filter the invalid information. The X_1_ is output of the multi-head self-attention mechanism and the X_2_ is the output of the feedforward neural network X_2_. By concatenating the X_1_ and X_2_ in the Formula (7), which can thoroughly combine the semantic dependencies captured by the Multi-Attention mechanism and the local features captured by the feedforward network; It can limit the information effectively by concatenating the feature vector X_3_ in the Eq (8–9) so that the Gated Linear Unit (GLU) in Formula (10) can control the information inflow of the limited feature vector adaptively; By fusing X_2_ in Formula (10), it can retain the more low-level and high-level semantic features and filter irrelevant noise and redundant information. The SAG model can better capture the crucial feature information in the text through local and global feature learning.

### 3.4 BiGRU

**Fig 5:**
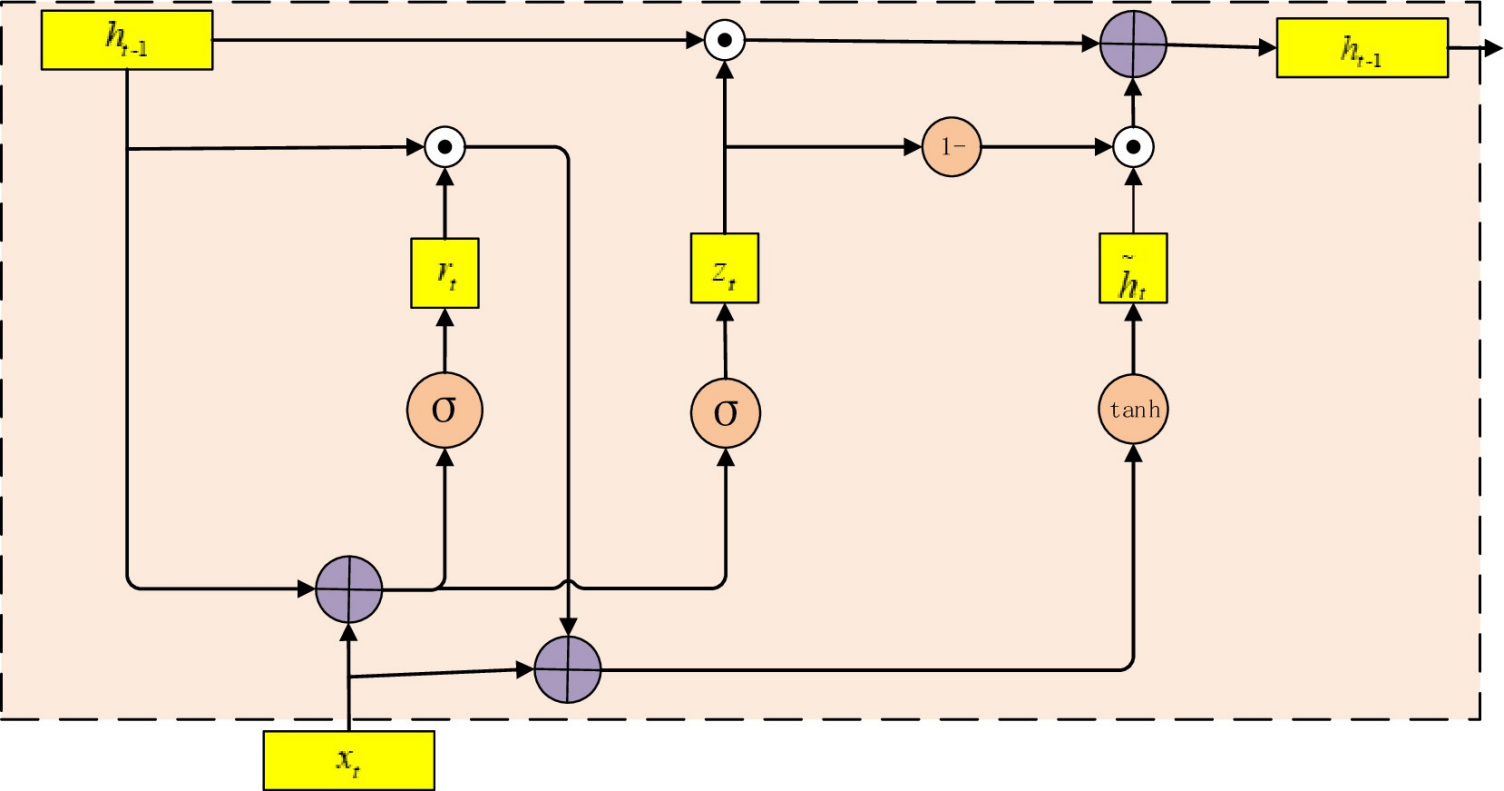
GRU module structure.

The API call sequence extracted during the dynamic execution of software is a long-time sequence. Using BiGRU to model the API call sequence can effectively avoid the problem of gradient disappearing and gradient explosion [40, 41], so it can effectively model the long-time API call sequence. The gated recurrent unit network (GRU) is an improvement of LSTM, which has the characteristics of fewer parameters and reduces overfitting. The GRU module structure is shown in Fig 5.

The GRU is mainly composed of an update gate and a reset gate. The update gate mainly controls the past information to the current state, and the reset gate mainly controls the previous state information to write into the current candidate set. Set the current moment t, and the GRU calculation Formulas (11–14) are as follows.


$$r_{t}=\sigma (\omega_{r}x_{t}+U_{r}h_{t-1}+b_{r})$$ (11)

$$z_{t}=\sigma (\omega_{z}x_{t}+U_{z}h_{t-1}+b_{z}),t\in [1,m]$$ (12)

$${\tilde{h}}_{t}=\tanh(\omega_{h}x_{t}+U(r_{t}*h_{t-1})+b_{h})$$ (13)

$$h_{t}=z_{t}*h_{t-1}+(1-z_{t})*{\tilde{h}}_{t}$$ (14)

Where σ is the sigmoid function, *ω*_*r*_, *ω*_*z*_, *ω*_*h*_ are input weight matrix, *b*_*r*_, *b*_*z*_, *b*_*h*_ are the bias value. *U*_*r*_, *U*_*z*_, U are state weight matrix, *r*_*t*_ is the reset gate, *z*_*t*_ is the update gate, ${\tilde{h}}_{t}$ is the candidate set of the current state, *h*_*t*_ is the final output.

Since GRU introduces an update gate and a reset gate structure, it is very effective for capturing long-distance features. When x_t_ input to the network unit, it is multiplied by its weight ω_z_. The same is done for h_t−1_, which keeps the information of the first t−1 units and is multiplied by its weight U_z_ as shown in Formula (12). These two results are added together, and then the sigmoid activation function is applied to compress the result to between 0 and 1. Update gates help the model determine how much information from the past needs to be passed on to the future. This is very powerful because the model can decide to copy all the information from the past and eliminate the risk of the gradient disappearing problem.

The reset gate is used by the model to determine how much past information is forgotten, as shown in Formula (11). ${\tilde{H}}_{t}$ is the current memory content, as shown in Formula (13), which will use the reset gate to store relevant information from the past. H_t_ is the final memory of the current time step, as shown in Formula (14). The model can learn to set the vector z_t_ close to 1 and retain most of the previous information. Since it will be close to 1 at this time step, (1− z_t_) will be close to 0, which will ignore most of the current information.

**Fig 6:**
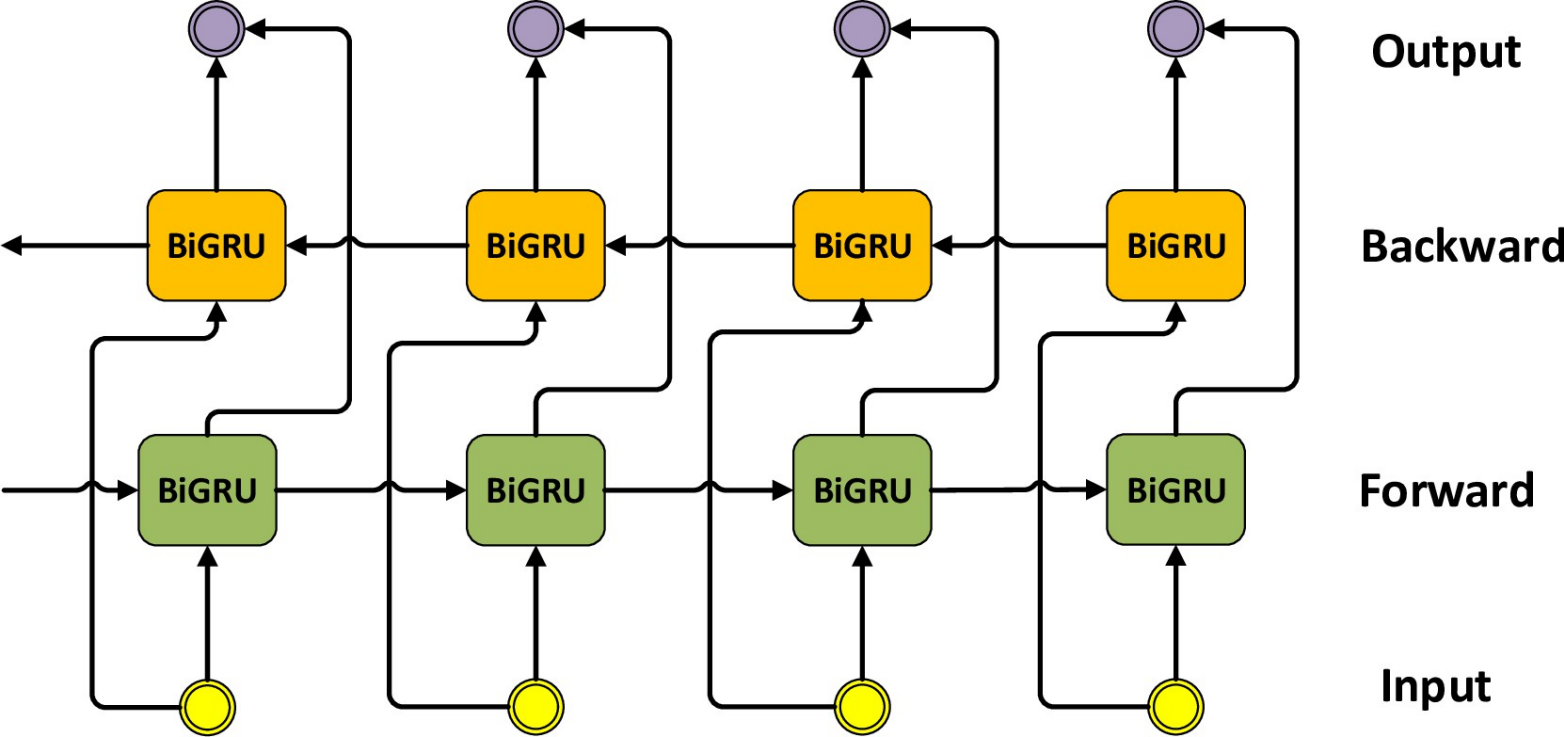
BiGRU module structure.

The BiGRU consists of a forward GRU and a backward GRU, and its backward sequence captures the information to make more accurate judgments. The BiGRU consists of two GRUs in different directions, as shown in Fig 6. At the moment t, the specific calculation formulas of BiGRU are shown in the Eqs (15–17). The final output of the BiGRU is denoted by H_t_ = [h_1_, h_2_,…,h_t_].


$$\vec{h_{t}}=GRU\;(h_{t},\vec{h_{t-1}})$$ (15)

$$\overset{\leftarrow }{h_{t}}=GRU\;(h_{t},\overset{\leftarrow }{h_{t-1}})$$ (16)

$$h_{i}=[\vec{hi},\overset{\leftarrow }{hi}]$$ (17)

BiGRU has a natural advantage for long-distance feature extraction. The CNN is limited by the receptive field of the convolutional kernel for extracting long-distance features. Increasing the size of the convolutional kernel and the depth of the network can increase the long-distance feature-capturing ability. However, it is still not as good as BiGRU. For Transformer, its long-distance feature capture capability is mainly affected by the number of multi-heads. The more multi-heads, the stronger the long-range feature capture capability of the Transformer.

## 4. Experiment and analysis

### 4.1 Experimental environment

Experimental configuration: The operating system is Ubuntu20.04 Server Edition, the processor is Intel Xeon Silver 4210, the memory is 64.0GB, the GPU is NVIDIA RTX A5000 16GB, and the programming language is Python3.9. The learning framework is Pytorch 1.11.0.

### 4.2 Training and test set

The experimental dataset in this paper comes from the training set provided by Alibaba cloud security malicious program detection data. Since the test set provided by the official data is not labeled, the data of the test set is discarded. We take the training set provided by the official as the total data sample and divide it into the training set and the test set according to the ratio of 4:1. The data comes from the API instruction sequence recorded after the desensitized Windows binary executable (PE file) is simulated in the sandbox program.

After the analysis of the training data, we found that the normal category of samples in the data set is the largest, accounting for 35.85%. Infectious virus is the largest type of malicious sample, accounting for 30.89% of the total samples. The number of worms is the least in the total sample, accounting for only 0.72%. The quantity distribution in the data set is shown in Table 4, which shows that the quantity of different samples in the data set is not balanced. We analyzed 295 different APIs in the dataset.

**Table 4:** Different category in the dataset.

Label	Category	Total
0	normal	4978
1	Ransomware virus	502
2	Mining program	1196
3	DDoS	820
4	Worms virus	100
5	Infectious virus	4289
6	Backdoor program	515
7	Trojan program	1487

The details of the training set after GNGS data preprocessing are shown in Table 5. Compared with the original data set, the new data set is generated with more balanced minority categories. It avoids the edge distribution of the minority categories, and the model training will not focus on the majority categories features while discarding the learning of the minorities features.

**Table 5:** Different category in the new dataset.

Label	Category	Total	percent
0	normal	5200	16.1%
1	Ransomware virus	3100	9.6%
2	Mining program	4300	13.3%
3	DdoS	3700	11.4%
4	Worms virus	3200	9.9%
5	Infectious virus	4900	15.1%
6	Backdoor program	3350	10.3%
7	Trojan program	4600	14.2%

### 4.3 Evaluation criteria

In Table 6 Confusion matrix [42, 43], true positive (TP) is the number of normal samples predicted as normal. True negative (TN) is the number of abnormal samples predicted as abnormal; False negative (FN) is the number of normal samples predicted as abnormal; False Positive (FP) is the number of abnormal samples predicted as normal.

**Table 6:** Confusion matrix.

Situation	Predict result	
	Positive	Negative
True	TP	FN
False	FP	TN

The evaluation indicators include the Accuracy (accuracy rate), Precision (precision rate), Recall (recall rate), and F1-score whose calculation formula are shown in the Eq (18–21).


$$Accuracy=\frac{TP+TN}{TP+FN+FP+TN}$$ (18)

$$Precision=\frac{TP}{TP+FP}$$ (19)

Recall=TPTP+FN (20)

$$F1-score=\frac{2*P*R}{P+R}$$ (21)

For the multiple classification problems, Literature [44] adopted the added weight based on the Macro ℱ1value calculation method, which is the same evaluation model as Chai et al. [45], whose precision, recall, and F1 value are with the weight by adopting the weighted mean, calculate evaluation indicators of each category, Taking Precision as an example, its calculation Formula is shown in (22). However, the evaluation method can’t reflect indicators of minority categories and will make the experimental results higher. For example, the experimental result of the minority category is very low, but the overall experimental result is still very high.

In this paper, the number of data correctly classified is divided by the total number of data to obtain the Accuracy of the multi-classification tasks, whose Formula is shown in (23). The Precision, Recall, and F1-score of each category are calculated respectively, and then these values were added and averaged to obtain the evaluation criteria of multi-category tasks. Taking Precision as an example, its calculation Formula is shown in (24).


$${\mathrm{Precision}}_{\mathrm{weighted}}=\sum_{i=1}^{n}w_{i}(\frac{TP_{i}}{TP_{i}+FP_{i}})$$ (22)

$$Accuracy_{multi}=\frac{\sum_{i=1}^{n}TP_{i}}{\sum_{i=1}^{n}\left(TP_{i}+FP_{i}\right)}$$ (23)

$$Precision_{multi}=\frac{\sum_{i=1}^{n}P_{i}}{n}$$ (24)

### 4.4 Parameter setting

The different hyperparameters settings will affect model convergence speed and experimental results. The configuration of hyperparameters in this experiment is shown in Table 7. The GSB model optimized the experiment parameters by the Adam optimizer, which can adjust the learning rate and achieve a good effect through some experiments.

**Table 7:** The configuration of parameter.

Parameter name	Value
BatchSize	512
Input_dim	32
LearningRate	0.0001
Dropout	0.5
Attention_head	4
FeedForward_hidden_size	64
BiGRU_hidden_size	64

### 4.5 Experimental results

We proposed the GNGS model for processing the unbalanced data and the SAG-BiGRU model for malware detection. The GSB model experimental results for multi-classification malware detection are shown in Table 8.

**Table 8:** Comparison of eight-classification experimental results.

Category	Precision	Recall	f1-score
Normal	0.95	0.98	0.97
Ransomware virus	0.86	0.91	0.89
Mining program	0.92	0.94	0.89
DDoS	0.83	0.85	0.79
Worms virus	0.81	0.83	0.87
Infectious virus	0.93	0.93	0.91
Backdoor program	0.87	0.85	0.91
Trojan program	0.91	0.93	0.94

The GSB model algorithm studies the distribution of the minority categories to avoid edge distribution problems. From the experimental results, each category is ideal. There is no very low accuracy for minority categories, indicating that the model has a good effect on the feature extraction of datasets. The precision of the normal category is relatively high, which can reach 95%, mainly because the proportion of normal training samples is large, and the model extraction features are more abundant. For the detection of minority categories, the precision of half categories can exceed 90%, and the model’s performance for malware detection of minority categories can achieve the expected effect. But the precision for Worms virus and DDoS is not high, because there are too few attack samples for Worms virus and DDoS categories in the original dataset, the training features extracted by the model are not rich enough, and the generalization ability is not enough, resulting in the test precision is not high.

### 4.6 Experimental discussion

This paper proposed the GNGS and SAG-BiGRU network for malware dynamic detection, which achieved an accuracy of 88.7% for the eight-classification and separated malicious software from benign software for the binary classification detection, which achieved an accuracy rate of 97.6%. Our model is compared with the other current studies, as shown in the figure.

#### a) Compared with current studies on the Alibaba Cloud dataset

**Fig 7:**
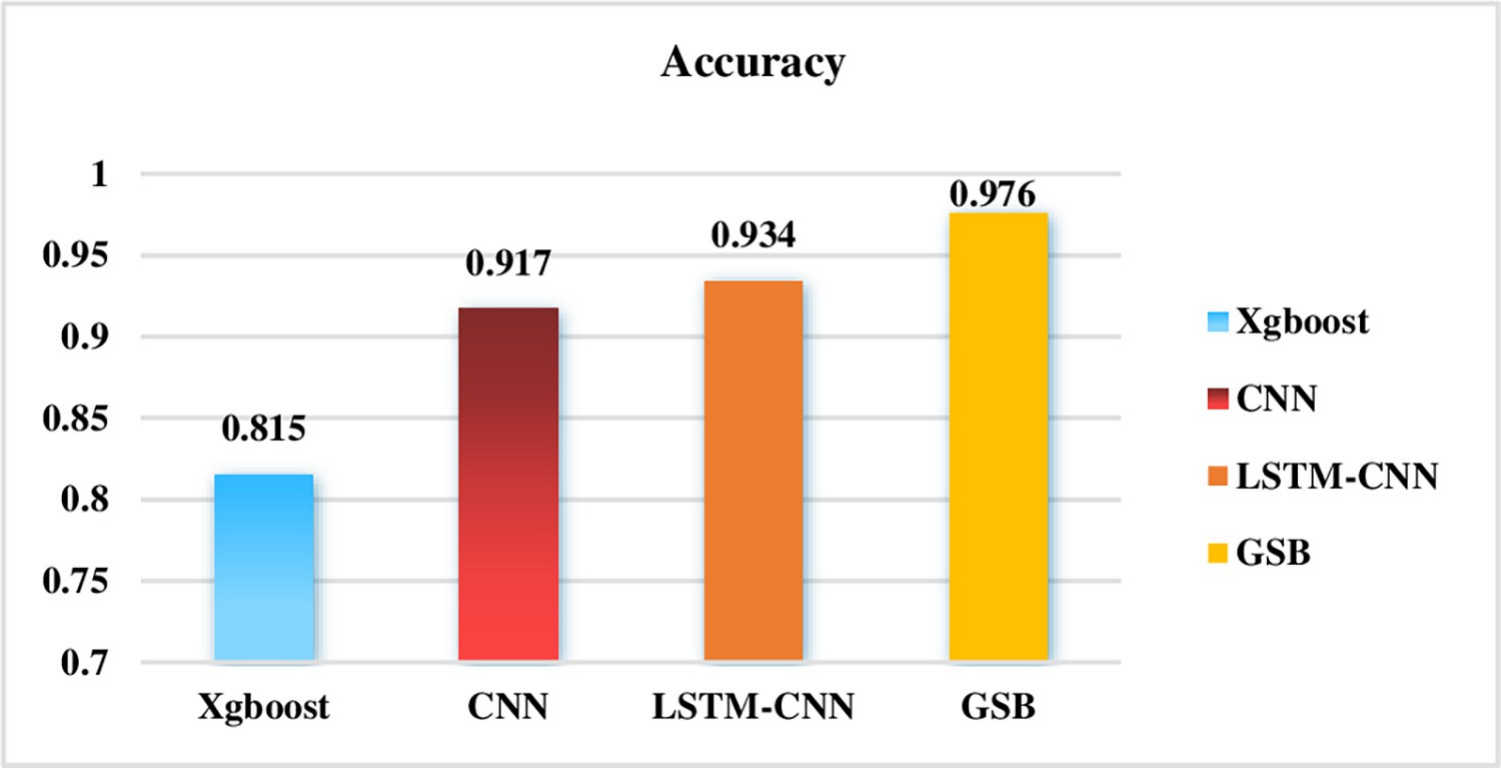
Accuracy of different algorithms in binary classification on Alibaba Cloud data set.

As for the study on the Alibaba Cloud dataset as shown in Fig 7, the current researches, Literature [46] and Literature [13] only conduct binary classification and can’t do the multi-classification research of malware. They only conducted de-duplication of the dataset and used deep learning CNN-LSTM algorithm for feature extraction to perform binary classification. Our GSB model performs better than CNN-LSTM binary classification, and also performs well for multi-classification of the minority categories.

The CNN model outperforms the Xgboost in terms of accuracy, and Xgboost needs to extract relevant features manually to achieve better results, besides, it needs more time and cost in actual use. The GSB model performs better than CNN, mainly due to the inevitable problem of forgetting when the sequence is too long. Of course, two-way BiGRU can alleviate such forgetting phenomenon to a certain extent, and the Transformer model has better semantic feature extraction and long-distance feature capture capabilities.

The main advantage of the SAG model based on Transformer is its excellent performance in processing long sequence data, especially for large dataset. Compared to traditional RNN or CNN, SAG model has better parallelism and shorter training times, which also has the ability to learn dependencies between sequences through self-attention mechanisms.

BiGRU has a natural advantage for long-distance feature extraction. The CNN is limited by the receptive field of the convolutional kernel for extracting long-distance features. Increasing the size of the convolutional kernel and the depth of the network can increase the long-distance feature-capturing ability. However, it is still not as good as BiGRU.

#### b) The GSB model for multi-classification on the Alibaba Cloud dataset

Most studies focus on majority categories in the whole data set and neglect to pay attention to the detection rate of the minority categories. However, these minority attacks usually cause more damage to the network than common attacks, so we should pay more attention to the detection of minority attacks.

In this paper, the deep learning model methods are used to explore the advantages of the GSB model compared with the common machine learning and deep learning methods such as Random Forest (RF), as shown in Table 9, the GSB model is superior to the traditional machine learning models.

**Table 9:** Comparison of eight-classification with different algorithms on the Alibaba Cloud dataset.

Model	Accuracy	Precision	Recall	F1-score
RF	0.687	0.690	0.512	0.576
BiGRU	0.722	0.472	0.502	0.512
Smote-BiGRU	0.768	0.686	0.612	0.585
SAG+BiGRU(SB)	0.784	0.613	0.576	0.492
Smote-SAG+BiGRU(SSB)	0.816	0.723	0.668	0.618
GNGS+SAG(GS)	0.862	0.858	0.866	0.862
GNGS+SAG-BiGRU(GSB)	0.887	0.885	0.902	0.896

We propose the GNGS model for processing unbalanced data and the SB model for malware detection. The accuracy of the GSB has a great improvement compared to the SB model, which proves that Gaussian noise generation strategies obtain a positive effect on processing unbalanced data.

The GSB model considers the extraction of temporal sequence features and long-distance dependency information compared with GS models, and the model explores whether the BiGRU module has an impact on classification results in terms of its role in preserving data temporal sequence features and long-distance dependency information. GRU is naturally suitable for processing temporal sequence data. Even if there is a gating mechanism, the problem of forgetting will inevitably exist when the sequence is too long, but the BiGRU can alleviate such a forgetting phenomenon to some extent. The SB model has better feature extraction ability and long-distance feature capture ability compared to the BiGRU model, and the self-attention mechanism gives the model better attention. By allowing it to focus on crucial relevant sequences of API calls, it can better solve the problem of forgetting. The Self-Attention with Gate mechanism (SAG) can carry out the key feature extraction and filter irrelevant noise information, which has better parallelism and shorter training time when dealing with large scale data, which also has the ability to learn dependencies between sequences through self-attention mechanisms.

The GSB model has a good effect on multi-classification detection compared to the SB and SSB model. Solving the unbalanced classification problem from the data level can make the classification model sensitive to unbalanced data. The Smote resampling method is often used to deal with category imbalances, but by comparing experimental results on datasets, GNGS method is more effective in improving the recognition rate of the model to the minority samples, and the training model will pay more attention to the useful minority category features. Smote algorithm is sensitive to noise and doesn’t have good effect for large-scale samples, and the synthetic sample is easy to be in the majority categories sample areas, forming noise samples. Finally it causes the overfitting of the model to reduce the precision of malware detection. We proposed the GNGS algorithm to deal with unbalanced minority attack data categories. GNGS algorithm is not only used for clustering, but also for estimating probability density. More importantly, the GNGS algorithm can generate new sample points and increase the robustness and generalization ability of the model. It shows that GNGS has a good effect on the unbalanced processing of data sets and is more reasonable for the feature extraction and the learning for the model.

#### c) The GSB model for multi-classification on NSL-KDD dataset

We used the GSB model for multi-classification on the NSL-KDD dataset. The NSL-KDD dataset is shown in Table 10, which is a new dataset generated from KDD_cup99 [47, 48].

**Table 10:** The details of the NSL-KDD dataset.

Type	KDDTrain		KDDTest	
	Record	Percent	Record	Percent
Normal	13449	53.38%	9711	43.07%
Probing	2289	9.08%	2421	10.73%
R2L	209	0.83%	2576	12.21%
U2R	11	0.04%	200	0.89%
DOS	9234	36.65%	7636	33.08%
Total	25192		22544	

The details of the training set after GNGS processing are shown in Table 11. We obtained a more balanced training dataset, especially for the minority categories R2L and U2R, which are more balanced after processing.

**Table 11:** The details balancing training data after GNGS processing.

Type	Record	Percent
Normal	10711	20.38%
Probing	10421	19.83%
R2L	10576	20.13%
U2R	10200	19.41%
DOS	10636	20.24%
Total	52544	

**Fig 8:**
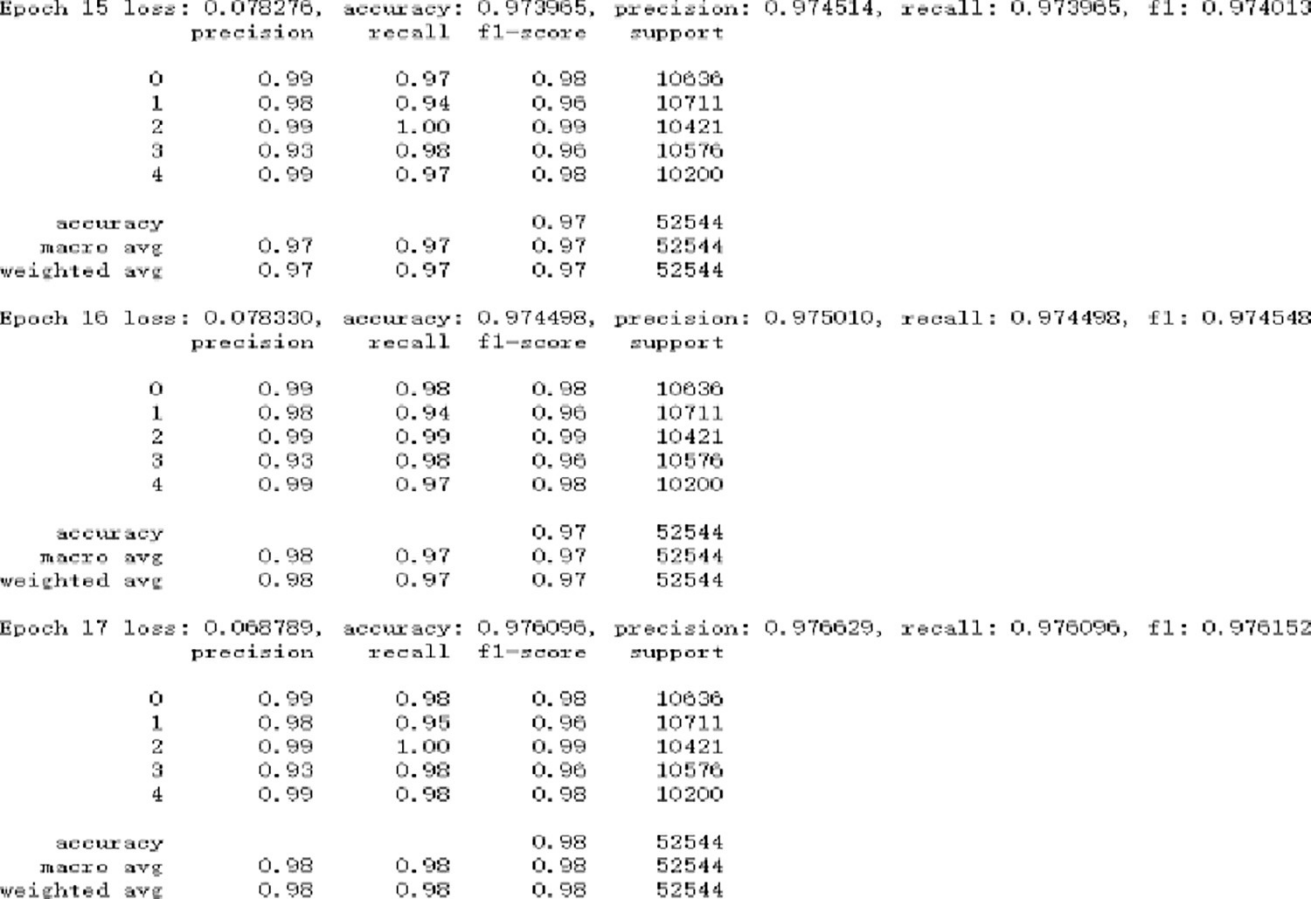
GSB model experimental results on the NSL-KDD.

After the GNGS processing, the GSB model experimental results on the NSL-KDD, as shown in Fig 8. The precision of the minority categories is more than 90% on the NSL-KDD dataset, which is much better than other algorithm models for the multi-classification.

The GSB model is much better than the RSSB model, as shown in Table 12, which produces a new dataset by using the Random undersampling and Smote oversampling algorithms that are popular data unbalanced sampling method. It can be seen that GNGS has a good effect in processing unbalanced data categories and the importance of processing unbalanced datasets for algorithm models.

**Table 12:** Comparison of five-classification with different algorithms on the NSL-KDD dataset.

Model	Accuracy	Precision	Recall	F1-score
Random-Smote+ SAG-BiGRU (RSSB)	0.834	0.753	0.671	0.696
GNGS+SAG (GS)	0.968	0.967	0.966	0.967
GNGS+SAG-BiGRU (GSB)	0.978	0.976	0.975	0.975

**Fig 9:**
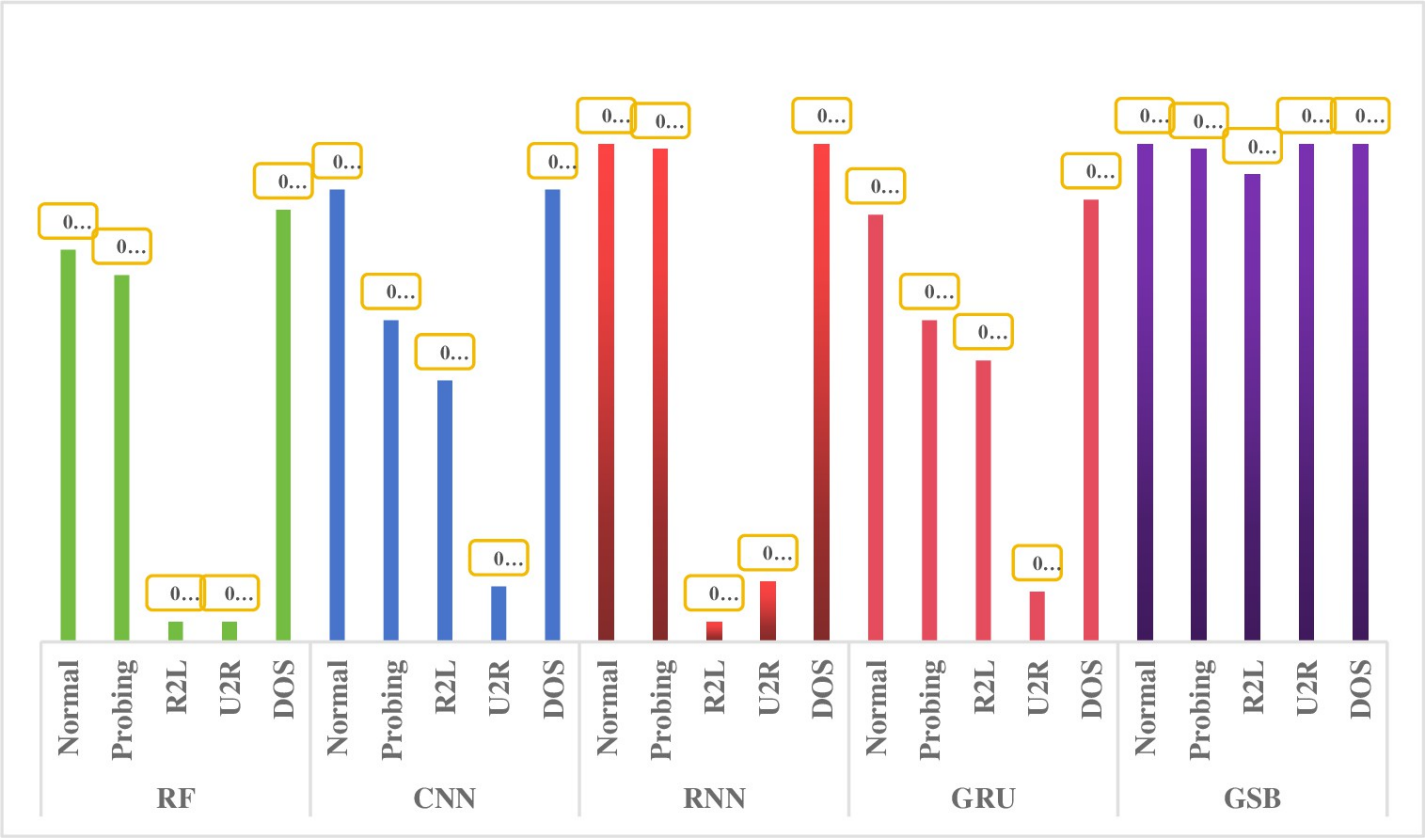
Accuracy of different algorithms model in five classification on the NSL-KDD dataset.

It can be seen that the GSB model is better than other algorithm models, as shown in Fig 9, especially for the detection of minority categories. Other research literatures [49–52] are difficult to detect the categories of U2R and R2L on the NSL-KDD dataset. Literature [53] proposed the conditional Generative Adversarial Networks (cGAN) to deal with the data unbalance, but the pricision is not high for the U2R and R2L categories. It can be seen that the GSB model has great advantages on the NSL-KDD dataset.

The shortcomings of the GSB model applied to the NSL-KDD dataset are mainly due to the small number of categories samples in the dataset, especially for U2R and R2L categories. Although the detection effect of U2R and R2L on the test set of the model can reach 90%, it is still hoped to further improve.

## 5. Conclusion

Malware detection and classification tasks from a dynamic analysis perspective, the study proposed the Gaussian Noise Generation Strategy (GNGS) algorithm to solve the problem of unbalanced distribution of data categories. The GNGS algorithm can generate new sample points and increase the robustness and generalization ability of the model. The training model will pay more attention to the useful minority category features, which is much better than other sampling methods, so that the precision of minority categories is much higher than the other research, and the accuracy of the majority categories is even up to 95%.

We proposed the Transformer deep learning models to learn the temporal sequence features and used the Self-Attention with Gate mechanism (SAG), which can effectively filter irrelevant noise information and extract the key features. The main advantage of the SAG model based on Transformer is its excellent performance in processing long sequence data, especially for large dataset models. Compared to traditional RNN or CNN, Transformer has better parallelism and shorter training times when dealing with long text, which also has the ability to learn dependencies between sequences through self-attention mechanisms. The GSB model also shows that the accuracy for the minority categories is close to 90% on the NSL-KDD data set, which is much better than other algorithm models for multi-classification.

The shortcoming of the paper is that we need more attack samples of the minority attack categories in the dataset, and the generalization ability of the training model for the minority attack categories is insufficient. It is hoped that we can continue to improve it in the future.

## Supporting information

S1 File (DOCX)
