# Supplementary material for: GSB: GNGS and SAG-BiGRU network for malware dynamic detection
Source: PLoS One. 2024 Apr 18;19(4):e0298809. doi: 10.1371/journal.pone.0298809 (PMC11025902; doi:10.1371/journal.pone.0298809)
Supplement: S1 File — (DOCX) [file pone.0298809.s001.docx]

Potential Data Availability statement for use

"All relevant data are available at https://tianchi.aliyun.com/dataset/dataDetail?dataId=137262 and within the manuscript'sSupporting Information files."

Zhanhui Hu, Guangzhong Liu, Xinyu Xiang, Yanping Li, Siqing Zhuang

2024.3.2
